# Cervical Chylous Fistula After Lymphadenectomy for Differentiated Thyroid Carcinoma: Anatomy, Risk Factors, and Management Strategies—A Narrative Review

**DOI:** 10.3390/jcm15207784

**Published:** 2026-10-09

**Authors:** Ovidiu Fabian, Emilia Maria Pătruţ, Flaviu Mureşan, Sergiu Neciu, Gabriel Emil Petre

**Affiliations:** 1 Department of Surgery, Iuliu Haţieganu University of Medicine and Pharmacy, 400012 Cluj-Napoca, Romania; 2 4th Surgical Clinic, CF Hospital Cluj-Napoca, 400015 Cluj-Napoca, Romania

**Keywords:** chylous fistula, thoracic duct, anatomical variations

## Abstract

**Background**: Cervical chylous fistula is a rare but potentially severe complication of laterocervical lymphadenectomy associated with thyroidectomy for thyroid carcinoma with lateral lymph node metastases. It occurs predominantly during level IV dissection in the proximity of the jugulo-subclavian confluence. Injury to the thoracic duct or the right lymphatic duct can generate persistent lymphorrhagia, leading to severe electrolyte imbalances, hypoproteinemia, and lymphopenia. The anatomical variability of the terminal portion of the thoracic duct justifies a comprehensive analysis of prevention and management strategies. **Methods**: A structured narrative analysis of data on thoracic duct anatomy, cervical compartment classifications, and therapeutic options was performed using a database search paired with citation tracking. Intraoperative identification techniques, ligation methods, and conservative and interventional strategies were critically evaluated. **Results**: Chylous fistula is mainly associated with left lower lateral compartment dissection (level IV) and cervical reinterventions. Daily drainage output is the central criterion for therapeutic decision-making, operationalized here through a comparative matrix and a stepwise algorithm. Low-output fistulas frequently respond to conservative treatment (hypolipidemic diet, medium-chain triglycerides, octreotide, or negative pressure). High-output fistulas require surgical reintervention with thoracic duct ligation (potentially associated with tissue adhesives or muscle flaps), video-assisted thoracoscopy, or percutaneous embolization. **Conclusions**: Prevention through systematic anatomical identification of the thoracic duct during level IV dissection, intraoperative verification of its integrity, and the application of a flow-based therapeutic algorithm drastically reduce morbidity and optimize the management of cervical chylous fistula.

## 1. Introduction

Chylous fistula is a rare complication of lymphadenectomy associated with thyroidectomy for thyroid carcinoma, with a frequency between 0.5% and 8% [1,2,3,4], but the complication is described even after thyroidectomy with minimally invasive lymphadenectomy [5].

The incidence is significantly lower in central compartment lymphadenectomy, occurring in fewer than 0.5% of cases, compared to lateral compartment lymphadenectomy where rates range from 0.5% to 8% [1,2,3,4,5]. Most fistulas occur on the left side due to the anatomy of the thoracic duct, which is larger, more fragile, and highly exposed in this region, yielding an incidence of 2% to 8% in left lower lateral dissections [1,2,3,4]. Although much rarer, fistulas can also occur on the right side due to damage to the right lymphatic duct, with reported rates ranging between 0.5% and 2% of right-sided lateral neck dissections [6]. The risk increases in the case of reoperations, the presence of bulky or infiltrative adenopathies at level IV (supraclavicular), and a history of cervical radiotherapy. This factor is usually associated with other types of cancer, not thyroid cancer.

To ensure clinical precision, a clear terminological distinction must be established between lymphatic complications. Chylous fistula (or chyle leak) refers specifically to the extravasation of lipid-rich, milky chyle resulting from direct injury to the main thoracic duct, the right lymphatic duct, or their major terminal branches. In contrast, a lymphatic fistula (or lymphorrhea) involves the leakage of clear, lipid-poor lymph from severed low-caliber micro-vessels or minor nodal channels. If unevacuated, localized accumulation of this fluid leads to a lymphocele. While lymphatic fistulas can occur in both central and lateral compartments, a true chylous fistula is highly localized.

If not controlled quickly, chylous fistula leads to a continuous loss of lymph rich in lymphocytes and nutrients, with severe systemic consequences (malnutrition, hypoproteinemia, deficiency of fat-soluble vitamins, hypovolemia, hyponatremia, hypochloremia, hypocalcemia, and lymphopenia). Locally, extravasation of chyle causes an inflammatory reaction that causes inflammation and, in severe cases, necrosis of the skin flaps.

Fistula treatment can be conservative, surgical or through interventional radiology, each therapeutic modality having advantages and disadvantages.

The conditions that lead to the appearance of chylous fistula following lymphadenectomy for thyroid carcinoma and its treatment options will be discussed.

## 2. Literature Search Strategy

A comprehensive literature search was conducted in the PubMed/MEDLINE and Google Scholar databases up to April 2026. The initial search utilized combinations of the following medical subject headings (MeSH) and free-text keywords: “chylous fistula”, “thoracic duct”, “neck dissection”, and “thyroid carcinoma”. To ensure a balanced and thorough overview, a “snowballing” approach (citation tracking) was subsequently applied; the reference lists of the retrieved original articles, recent meta-analyses, and international clinical guidelines were manually screened to identify additional foundational and relevant studies. To minimize selection and publication biases, specific pragmatic criteria were applied. Inclusion criteria comprised peer-reviewed clinical studies (retrospective/prospective), systematic reviews, meta-analyses, and landmark case series focusing explicitly on terminal thoracic duct anatomy, risk factors for cervical lymphatic injury, intraoperative prevention techniques, or post-operative management algorithms in thyroid and head-and-neck surgeries. Exclusion criteria applied to publications in languages other than English or Romanian, studies focusing exclusively on thoracic or abdominal chylous complications (e.g., chylothorax secondary to esophagectomy or spinal surgery without cervical components), and abstracts from conferences lacking complete peer-reviewed datasets.

To ensure a critical and transparent evaluation of the therapeutic modalities synthesized in this review, the strength of supporting evidence was classified into three pragmatic levels based on study design, adapted from the Oxford Centre for Evidence-Based Medicine (OCEBM) criteria: High strength was assigned to interventions validated by systematic reviews, meta-analyses, or prospective comparative studies; Moderate strength was assigned to consensus driven by retrospective cohort studies, institutional guidelines, or well-designed multi-center case series; Low strength was applied to strategies supported predominantly by isolated case reports, small clinical series (<10 patients), or expert consensus opinions lacking structured data analysis.

Given the low incidence of cervical chylous fistula specifically following thyroid surgery, expanding the literature selection to include high-quality evidence from broader head-and-neck oncology and interventional thoracic surgery was methodologically required to provide a robust synthesis of current therapeutic success rates. However, the primary clinical focus of this narrative analysis remains strictly anchored within the framework of thyroid carcinoma management.

## 3. Type of Lymphadenectomy

**Figure 1:**
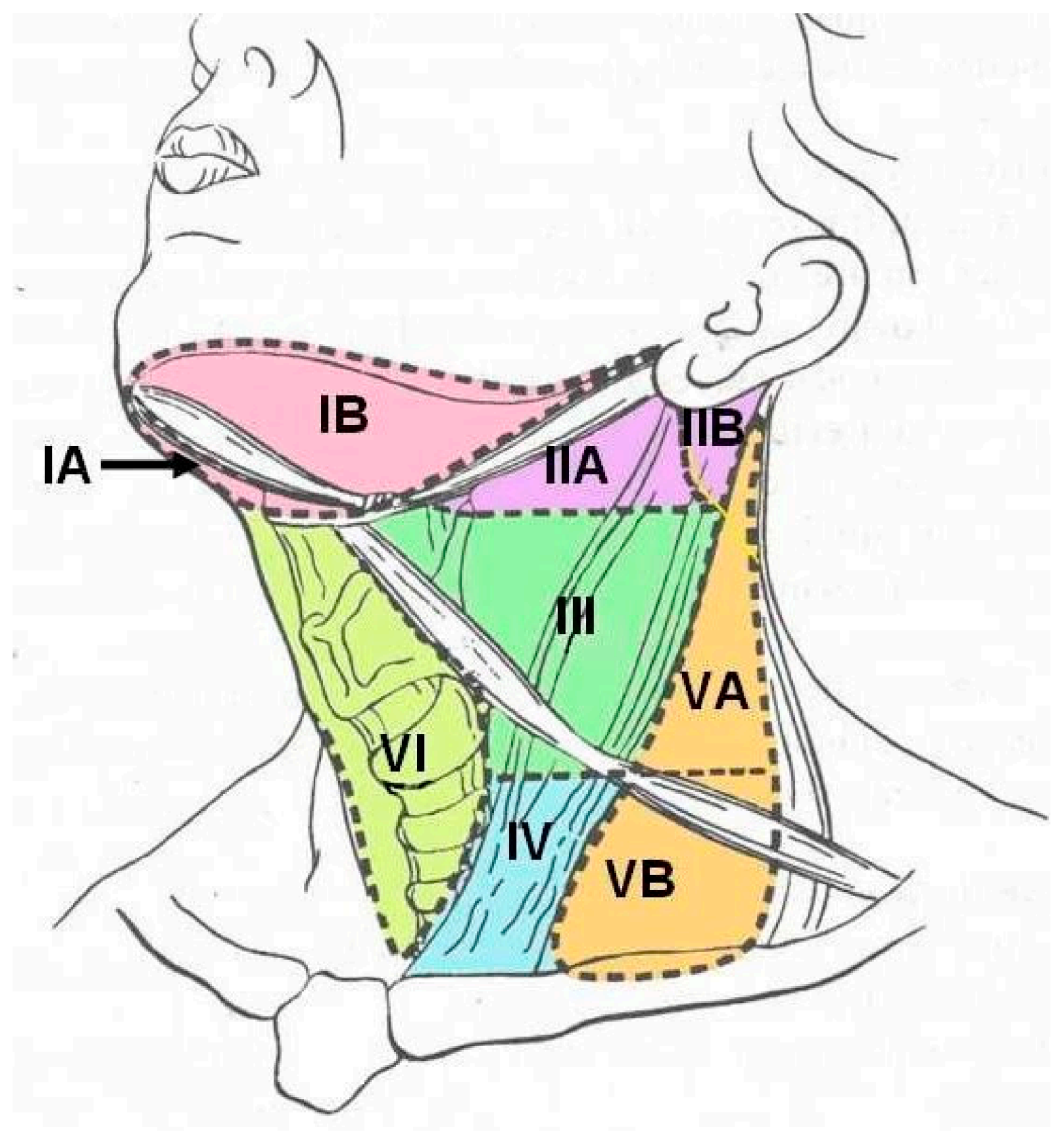
Schematic representation of cervical lymph node compartments (Levels I–VII) relevant to thyroid carcinoma dissection. Adapted from Harish, K. (2005), licensed under Creative Commons Attribution License (CC BY 2.0) [8].

The lymph nodes of the neck are systematized in 6 groups: group I includes the submental (IA) and submandibular (IB) nodes. Group II includes the jugular nodes located above the level of the hyoid (in relation to the accessory nerve. This group is divided into IIA and IIB). Group III includes the jugular nodes located caudal to the level of the body of the hyoid bone. Group IV includes the jugular nodes located caudal to the body of the hyoid bone up to the sternal manubrium. Group V includes the nodes of the posterior triangle of the neck (divided by the omohyoid muscle into VA—cranial and VB—caudal). Group VI includes the central nodes (pretracheal, prelaryngeal, paratracheal, perithyroid). Group VII includes the superior mediastinal nodes [7]. The retropharyngeal and parapharyngeal nodes are not included in this system. The discussed ganglion stations are schematized in Table 1 and Figure 1.

Cervical lymphadenectomy must be adapted depending on the pathology present and the degree of tumor dissemination. The operation must ensure the oncological objective, but also minimize risks, complications and sequelae. Functional lymphadenectomy aims to spare the structures of the neck [9,10,11], among which is the thoracic duct. Lymphadenectomy must be justified, adapted to the patient, the tumor and the suspicion of lymph node involvement, based on clinical examination, ultrasound and cytology [12].

The preoperative diagnosis can be confirmed cytologically or it can be only a suspicion. The clinical suspicion of thyroid carcinoma is strengthened based on the ultrasound criteria: nodules with microcalcifications, marked hypoechogenicity, irregular margins, rich central vascularity, and shape (taller than wide) combined in the TI-RADS score, while the high value of serum calcitonin points towards medullary carcinoma. Confirmation is obtained based on Fine-Needle Aspiration Cytology (FNAC), but this can have uncertain results. Sometimes the confirmation of malignancy is a pathological surprise after thyroidectomy [13]. Evaluation of the lymph nodes begins with ultrasound. Ultrasound criteria for the metastatic character of lymphadenopathies are: enlarged size, round shape, hypoechoic cortex, cortical thickening with absent or displaced hilum, loss of normal hilar architecture, hyperechoic foci, microcalcifications, cystic changes, capsular disruption, perinodal hyperechoic rings, matted nodes, or transcapsular blood flow [14,15,16]. Additional investigations (CT, biopsy) are sometimes necessary.

Cervical lymphadenectomy (neck dissection) in thyroid carcinoma can have a curative (therapeutic) or prophylactic role, depending on the presence of lymph node metastases detected clinically or through imaging. Preoperative establishment of the diagnosis of carcinoma, the type of carcinoma and the lymph node extension are essential for planning the primary operation. Other times the planning is done after histopathological confirmation or for residual or recurrent adenopathies.

In follicular and papillary carcinoma, lymphadenectomy is indicated when there are clinically or imaging-confirmed lymph node metastases [17,18,19]. Lymphadenectomy includes the compartments for which there are imaging arguments of the presence of lymph node metastases. When metastases are confirmed in the central compartment, but not confirmed in the lateral compartment, the question arises whether central lymphadenectomy should be completed with lateral compartment lymphadenectomy. For follicular carcinoma, prophylactic lateral lymphadenectomy is unjustified, as this carcinoma has predominantly hematogenous dissemination [20,21,22]. For papillary carcinoma, the criteria indicating an increased risk of occult metastases in the lateral compartment include some features of the central compartment metastases, some characteristics of the primary thyroid tumor, some demographic features of the patients, some molecular features, and some ultrasound features.

**Figure 2:**
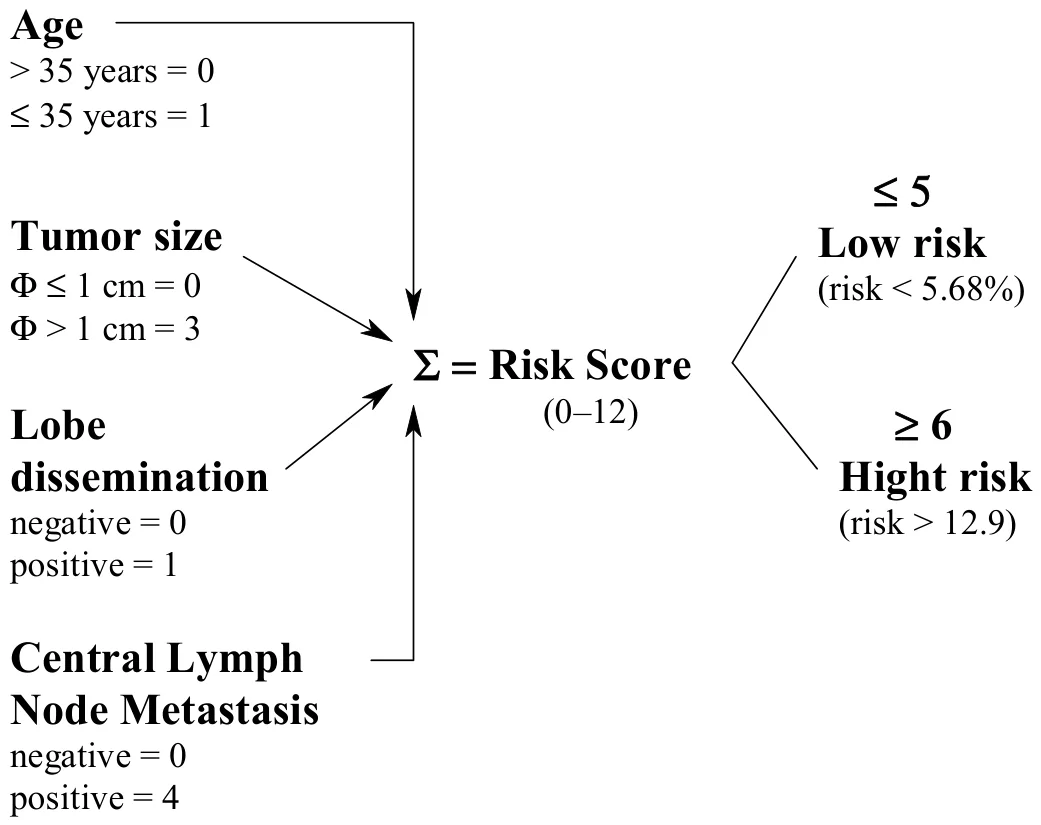
The score proposed by Guo et al. for assessing the risk of lateral compartment lymph node metastases [29]. Each parameter (age, tumor size, lobe dissemination, central lymph node metastasis) is quantified, and the score is obtained by summing the 4 parameters. The risk is considered low (<5.68%) for score values between 0 and 5 and high (>12.9%) for score values from 6 to 12.

The presence of more than 3–5 metastatic lymph nodes in the central compartment is a strong predictor of lateral involvement. Central metastases that show capsular rupture (loss of continuity of the nodal capsule on high-resolution ultrasound) increase the likelihood of invasion into the lateral nodal chains. Tumors located in the upper pole of the thyroid lobe have a higher risk of metastasis to the lateral compartment, including skipping the central compartment (skip metastasis). Tumors with a diameter > 1 cm, multifocal tumors, and tumors with extrathyroidal extension have a higher risk of lateral metastases. The BRAF V600E mutation is associated with increased aggressiveness and a higher rate of nodal metastasis, but its determination is not a common clinical practice. Young patients (under 45–55 years) or male patients may be at higher risk of extensive nodal involvement. High-resolution ultrasound may show the presence of hyperechoic areas in the lateral nodes or suspicious perinodal vascularity on Doppler ultrasound, which may suggest micrometastases [23,24,25,26,27,28]. Combining these criteria, Guo et al. proposed a score to assess the risk of lateral compartment lymph node metastasis in patients with papillary carcinoma [29]. The score is obtained by summing the values assigned to the following criteria: age (>35 years = 0, ≤35 years = 1), tumor size (≤1 cm = 0, >1 cm = 1), lobar dissemination (absent = 0, present = 3), capsular invasion (absent = 0, present = 1) and the presence of central compartment lymph node metastasis (absent = 0, present = 4). Score values between 0 and 5 indicate a low risk of lateral compartment metastasis, while values between 6 and 12 indicate a high risk—Figure 2.

In the case of medullary carcinoma, which has a high rate of nodal metastasis and does not respond to radioactive iodine treatment, surgery should ensure removal of the tumor and its nodal disseminations. Bilateral central lymphadenectomy is indicated when tumor diameter is ≥1 cm, in the presence of bilateral disease, in hereditary forms and when calcitonin exceeds 20 pg/mL. This involves removal of the nodes between the hyoid bone and the innominate vessels. Lateral lymphadenectomy is performed when there are ultrasound signs of metastases in the lateral compartment or when the presence of nodal metastases in the lateral compartment is confirmed by puncture and cytological examination [30,31,32].

## 4. Anatomical Variants of Thoracic Duct Termination

The thoracic duct is responsible for lymphatic drainage from the entire body, except for the right side of the head and neck, the right side of the chest, and the right upper extremity (which are drained by the right lymphatic duct). It originates in the cisterna chyli. It ascends between the aorta and the azygos vein anterior to the spine, then to the right of the spine. At the level of C7, it passes posterior to the esophagus and passes to the left behind the left carotid artery, the vagus nerve, and the left internal jugular vein. It then arches 3–4 cm above the clavicle and empties into the venous system at the junction of the left subclavian vein and the left internal jugular vein. It has unicuspid and bicuspid antireflux valves along its course. At the junction with the venous system, there is a bicuspid valve that prevents blood from refluxing into the lymphatic duct [1,33,34,35,36].

In addition to the anatomy mentioned above, there are variations regarding the path of the thoracic duct, whether it opens into the venous system on the left or right, and the manner of opening into the venous system. The variability of the anatomy of the terminal portion of the thoracic duct is one of the factors that lead to the risk of damage to the duct during lymphadenectomy and implicitly to the risk of chylous fistula. The variability is the consequence of the way the lymphatic system develops in intrauterine life.

**Figure 3:**
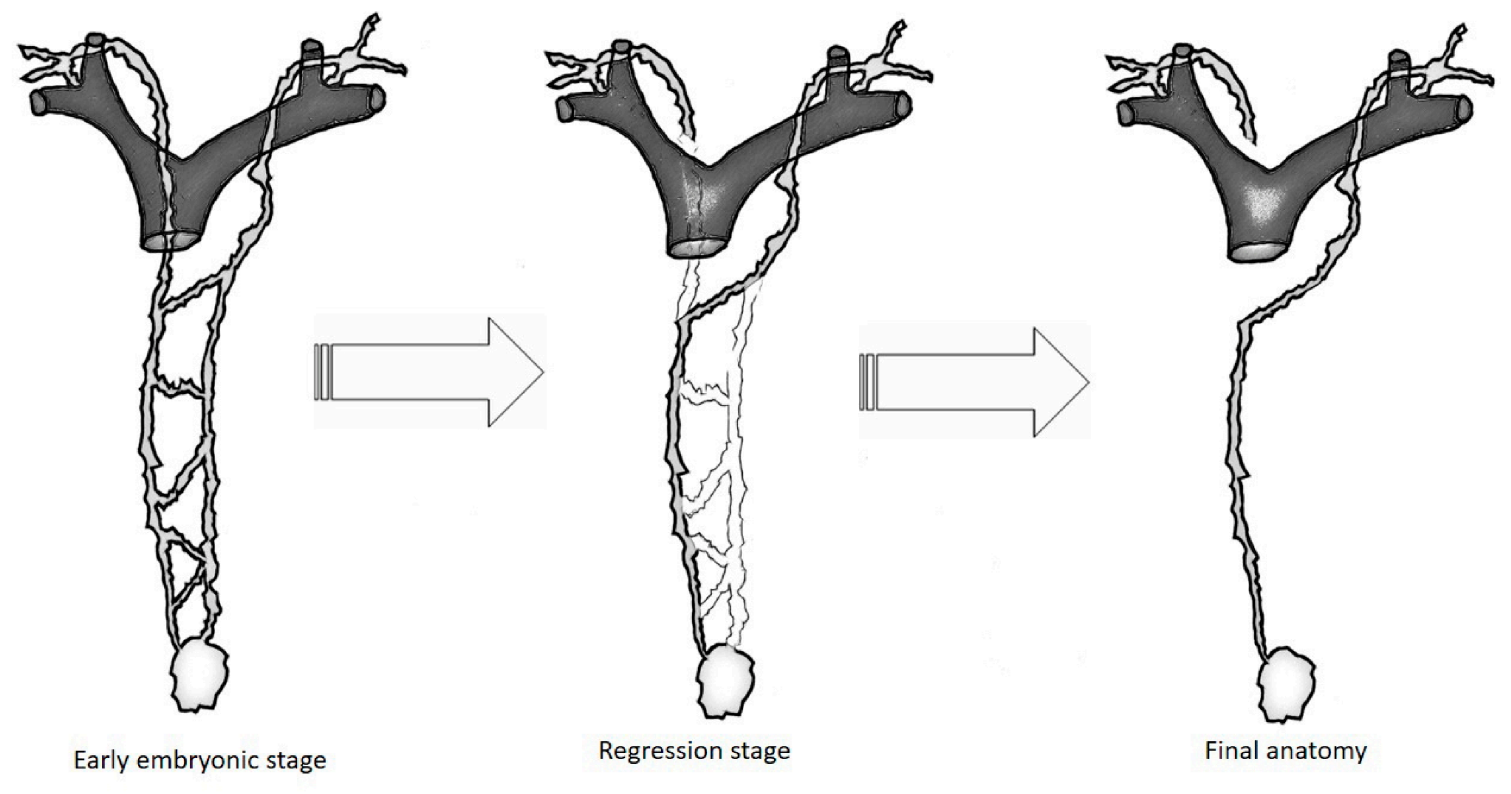
Development of the thoracic duct. Initially, 2 ducts (one on each side) depart from the cisterna chyli and are connected to each other by anastomoses. Through regression of the lower part of the left duct, the upper part of the right duct and the anastomoses (except for the right-left anastomosis at the T6-T4 level), the thoracic duct takes its final form with the lower portion located on the right, crossing the midline and the upper portion located on the left [35].

The formation of the lymphatic system begins in the 6th week of embryonic life. Initially, 2 buds appear from which 2 lymph sacs develop in the vicinity of the internal jugular veins. By the end of the embryonic period, 6 lymph sacs are formed: 2 jugular sacs, 2 iliac sacs, 1 retroperitoneal sac and the cisterna chyli. The lymphatic vessels develop similarly to the blood vessels and unite these sacs. The connection of the jugular sacs with the cisterna chyli is initially made through a system of bilateral trunks (on either side of the midline). The 2 trunks are interconnected through a system of anastomoses. By the disappearance of portions of the two trunks and most of the anastomoses, the thoracic duct is formed. The thoracic duct originates from the lower part of the right trunk, the upper part of the left trunk and a diagonal anastomosis at the level of the T4–T6 segments—Figure 3. In the early fetal period the lymph sacs transform into groups of lymph nodes. The exception is the upper part of the cisterna chyli, which remains a lymphatic collector. The most cranially located lymph node formed from the lymph sacs is the Virchow node and is located in the vicinity of the jugulo-subclavian venous junction [35].

Anatomical variants of the thoracic duct are common and can occur along its entire course, from origin to termination. The lymphatic sac from which the cisterna chyli develops can transform in several ways, leading to variants of the origin of the duct: the absence of the cisterna chyli (direct union of the lumbar and intestinal vessels into a duct) or the cisterna in the form of a network of lymphatic vessels. At the level of the thorax, both initial ducts can persist, leading to the existence of two thoracic ducts, or the anastomosis that crosses from right to left can regress, and the thoracic duct remains on the right until termination [1,35,36].

The transformation of the jugular lymph sacs also has variations, which lead to anatomical variants of the terminal portion of the thoracic duct. To these is added the termination on the right side, already mentioned above, and the existence of lymphatic tributaries that open right at the terminal portion of the duct.

Variants regarding the termination of the duct are those of surgical importance regarding the risk of chylous fistula occurring following cervical lymphadenectomy.

The thoracic duct may drain into the venous system of the neck on the right side, especially into the right subclavian vein or the right internal jugular vein. In rare cases, double thoracic ducts may drain into the venous system bilaterally, one on the left and one on the right side of the neck. Variability also affects the veins into which the duct opens. Although the typical opening is at the confluence of the internal jugular vein and the subclavian vein, the duct may also open into the left internal jugular vein, the left subclavian vein, the left brachiocephalic vein, the left external jugular vein, or even the left vertebral vein.

**Figure 4:**
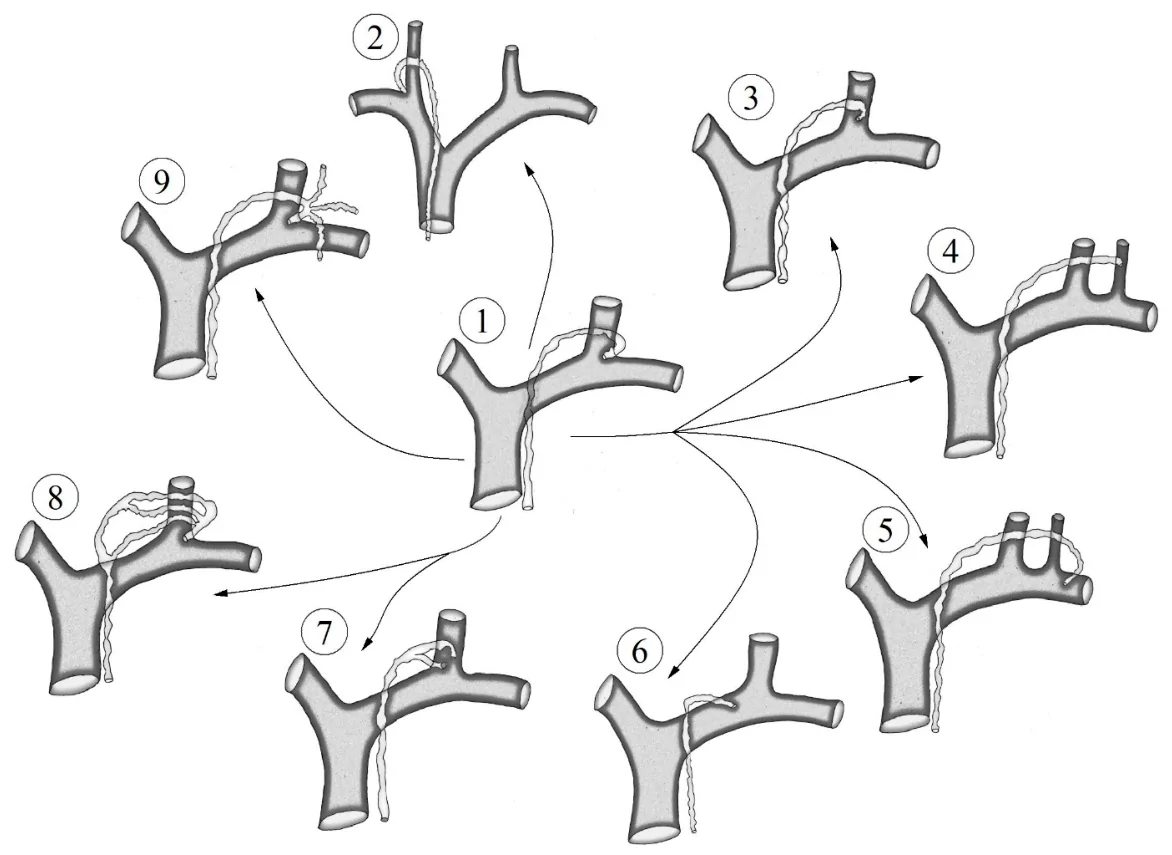
Variants of the terminal portion of the thoracic duct. 1. Typical variant—the duct opens into the left jugular-venous angle. 2–6. The duct ends in other veins: 2 in the right jugular-venous angle, 3 in the left internal jugular vein, 4 in the left external jugular vein, 5 in the left subclavian vein, 6 in the left brachiocephalic trunk. 7–8. The duct may have terminal ramifications: 7 there may be 2–3 branches that open separately into the venous system; 8 the ramifications unite into a single trunk. 9. In the terminal portion the duct has 3 tributaries: the left jugular trunk, the left subclavian trunk and the left bronchomediastinal trunk.

There may also be a branching of the terminal portion of the duct. The duct may divide into two or three branches. These branches may in turn open into different veins or may unite in a terminal duct. The duct may present segmental dilatations (moniliform appearance) or may have a wavy, arched appearance. The tributaries of the thoracic duct in the cervical region are the left jugular trunk, the left subclavian trunk, and the left bronchomediastinal trunk. These may open individually into the thoracic duct or through a common channel [34,35,36,37,38] (Figure 4).

In a meta-analysis, Plutecki proposes a classification of termination variants into six types depending on the vein in which the duct opens: type 1 (45.29%) in the left venous angle, type 2 (24.19%) in the left internal jugular vein, type 3 (16.32%) in the left subclavian vein, type 4 (1.51%) in the left external jugular vein, type 5 (1.02%) in the left brachiocephalic vein and type 6 (<1%) in the left vertebral vein or in the right jugulo-venous angle [38].

Along its entire length, the duct has bicuspid valves that prevent reflux and ensure the direction of lymphatic circulation. The segments between 2 valves define the lymphangions, respectively the morphological and functional units that, through the contraction of intrinsic muscle fibers, ensure the propulsion of the lymph. The presence of valves gives the duct a moniliform appearance. The portion of the duct located above the aortic arch and the cervical portion of the duct have more valves, located at shorter distances from each other, probably due to the higher pressure at these levels, which requires compartmentalization of the liquid column. At the level of the lymphovenous junction or on the last 10–30 mm of the duct there is a bicuspid valve that prevents blood reflux into the duct [39,40,41,42].

The diameter of the duct in the terminal portion varies between 2 and 5 mm [38,42], the average being 3.79 mm [42]. The diameter varies with pressure variations during breathing and with position, being larger during inspiration and in the Trendelenburg position and smaller during expiration and in the supine position [43]. In the terminal portion the thoracic duct describes an arc 3–4 cm above the clavicle [36]. Sometimes this arch is wider, reaching 7–8 cm above the clavicle [44].

The wall of the thoracic duct is composed of 3 layers: intima, media, and adventitia. The intima comprises a layer of endothelial cells and a subendothelial layer composed of a complex of smooth muscle and connective tissue. The media contains a few compact circular smooth muscle fibers, elastic fibers, and collagen fibers. The adventitia comprises a complex of connective tissue. The wall thickness in young adults is approximately 0.45 mm and decreases by approximately 20% in the elderly due to partial loss of smooth muscle [42].

From an operative standpoint, it is clinically counterproductive to attempt to link each micro-anatomical variation to a specific, unique mechanism of iatrogenic injury. Rather, the primary surgical risk does not stem from a single specific variant, but from the surgeon’s lack of awareness regarding this overall structural unpredictability. The critical safety factor is acknowledging that anomalous, multiple, or high-arched terminal branches can occupy the entire level IV clearance zone, which mandates a strategic shift toward active intraoperative verification and defensive dissection maneuvers rather than attempting to predict specific pathways [38,42].

## 5. Aspects of Surgical Technique

The duct has very thin walls and is a fragile structure, prone to rupture even with gentle handling. Damage to the duct can occur by sectioning, crushing, or tearing by traction on adjacent structures.

Lateral lymphadenectomy involves incision of the cervical fascia along the anterior border of the sternocleidomastoid muscle, followed by dissection of the neurovascular bundle, isolation of the internal jugular vein, and elevation of the adipose and lymphatic tissue along the vein. In the lower part (at the level of group IV), several structures must be protected: the phrenic nerve, the vagus nerve, the thoracic duct (on the left side) or the right lymphatic duct (on the right) [10].

Prevention of thoracic duct injury is achieved by identifying it and delicate dissection around it. The most important anatomical landmark is the angle between the internal jugular vein and the subclavian vein, where the duct opens into the venous system, but it should not be forgotten that there are anatomical variations. Several maneuvers can be used to identify the thoracic duct that help identify it. The Valsalva maneuver by increasing the pressure up to 30–40 cm of water for 15–30 s determines the turgor of the duct, making it easier to identify [36,45]. However, the maneuver is only effective when there is a thin layer of tissue over the duct through which a slight bulge can be observed. The maneuver cannot be maintained throughout the dissection, but it can be repeated several times to confirm the position of the duct. A thread or clip can be applied nearby for guidance. Dissection is not performed during the maneuver, on the one hand because the time is too short and on the other hand because the duct walls become even thinner and more fragile during the distension period. Much more effective is the identification of the duct by lymphography with indocyanine green. It can be performed by introducing the substance into the interdigital spaces of the feet or into the inguinal lymph nodes (under ultrasound guidance) immediately after orotracheal intubation, considering that the time required for the substance to diffuse to the level of the duct is 15–90 min [46]. The method allows visualization of the duct through a 5–10 mm layer of tissue. Once the duct is identified, the removal of adipose and lymphatic tissue is done, leaving a thin layer of tissue with a protective role. During the dissection, all visible lymphatic branches are ligated. Optionally, after checking the integrity of the thoracic duct, collagen sponges can be applied.

**Figure 5:**
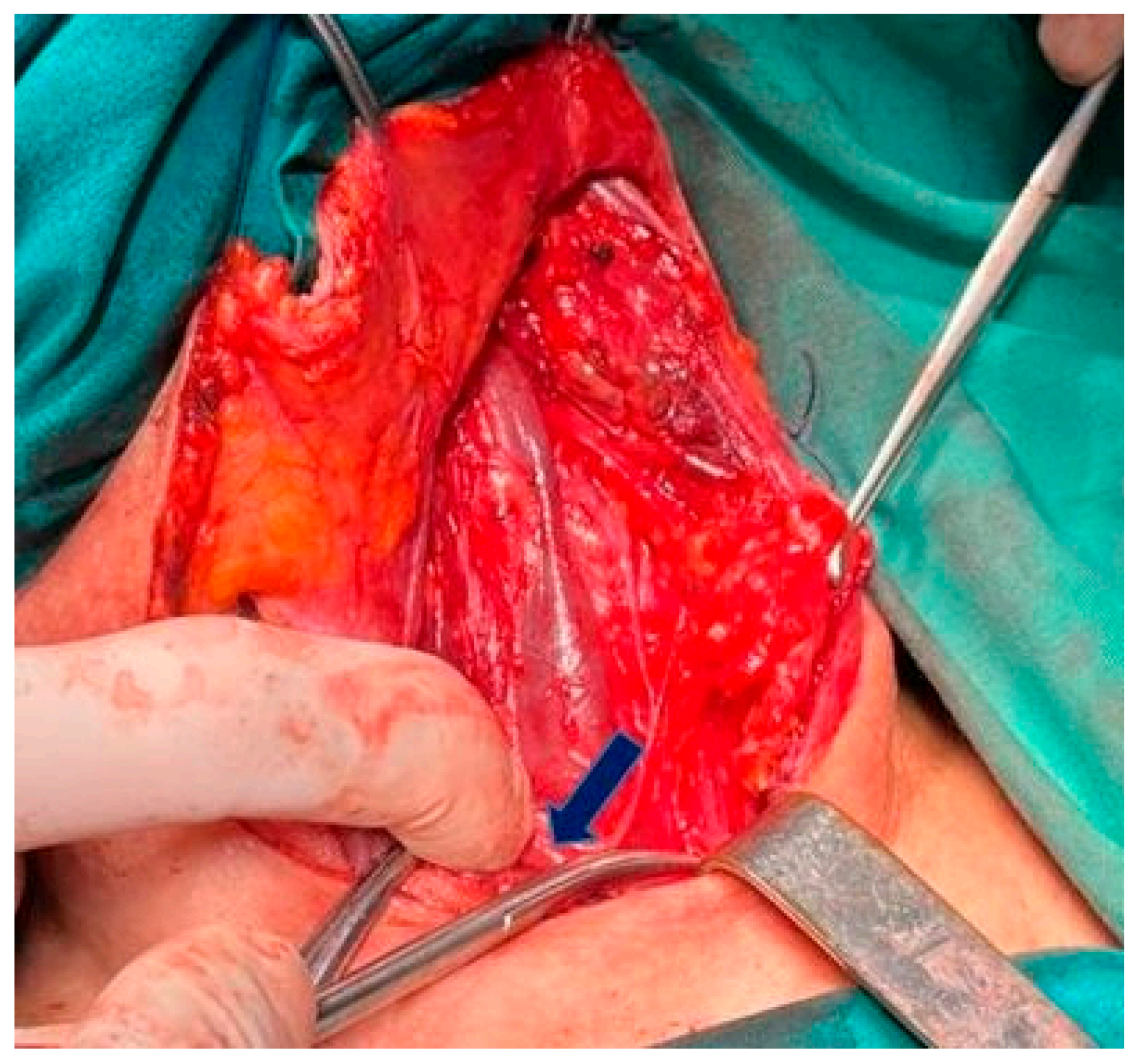
Intraoperative visualization of the thoracic duct in the venous angle between the internal jugular and subclavian veins.

Intraoperative verification of the integrity of the duct is done by placing the patient in the anti-Trendelenburg position (to relieve congestion of the vessels at the base of the neck), drying the surgical field and performing the Valsalva maneuver. In the case of a lesion, lymph drainage can be observed. If indocyanine green lymphography was used, a final examination in infrared light can highlight the leakage. Finally, drainage is placed with 2 tubes (one on each side) passed through the contraincision (Figure 5).

Recently, Liu et al. proposed a reverse dissection technique that aims to protect the thoracic duct and the right lymphatic duct and prevent chylous fistula [47]. The fascia covering the surface of the internal jugular vein and the clavicle is incised from superior to inferior, exposing the adipose tissue occupying the venous angle. The distal end of the adipose tissue mass is gently pulled and progressively removed from the venous angle. The lymphatic vessels present in this adipose tissue mass are dissected from distal to proximal. These vessels converge into the thoracic duct or the right lymphatic duct. The main trunk of the thoracic duct or the right lymphatic duct is protected, and those branches are ligated at their openings into the large ducts. The residual tissue is then meticulously cleaned by precise dissection.

From a practical surgical standpoint, it is clinically impossible to tailor a distinct dissecting technique for each specific anatomical variant of the thoracic duct due to its extreme unpredictability. Instead, awareness of these variations shifts the focus toward comprehensive tactical strategies, such as the reverse-sequence dissection proposed by Liu et al. [47]. Initiating the lateral neck dissection directly with level IV (the high-risk zone) offers two major clinical advantages:

1. It allows the surgical team to approach the most hazardous anatomical crossroads at the beginning of the procedure when focus and visual acuity are at their peak, minimizing technical errors due to fatigue.
2. By performing the level IV clearance early, any inadvertent micro-injury to an anomalous or fragile lymphatic branch has sufficient operative time (often 1–2 h during the remainder of the thyroidectomy and central dissection) to manifest as a visible chylous or lymphatic leak before skin closure, allowing for immediate intraoperative repair.

However, a critical limitation of this 2026 protocol lies in cases presenting with bulky, infiltrative adenopathies firmly fixed to the internal jugular vein confluence. In such hostile oncological environments, initial retrograde traction might paradoxically tear a fragile, hidden thoracic duct arch before proper vascular mobilization is achieved. Hence, this tactical approach should be carefully selected based on preoperative mobility mapping of the lymph nodes [47].

Regarding the extent of thyroid surgery for differentiated thyroid carcinoma, contemporary international guidelines (such as the American Thyroid Association (ATA) consensus) focus the therapeutic choice between Total Thyroidectomy (TT) and Thyroid Lobectomy/Hemithyroidectomy for low-to-intermediate risk tumors, while subtotal resections are no longer recommended due to oncological safety concerns. From a morbidity standpoint, a comprehensive bilateral procedure like TT is statistically associated with a wide spectrum of classic early and late surgical complications—most notably transient hypocalcemia, recurrent laryngeal nerve neuropraxia, and post-operative hematomas—which mandate strict preventive strategies and meticulous management protocols [48]. Within this broad landscape of thyroid surgical morbidity, complications involving terminal lymphatic channels and true cervical chylous fistulas represent a rare, highly specific subset that arises primarily when clearance is extended into the lateral compartments.

In modern endocrine surgery, the utilization of advanced energy-based devices, such as the Harmonic Scalpel (HS) and the LigaSure (LS) vessel sealing system, has become widespread alongside traditional suture ligation. Current literature indicates that both devices exhibit comparable safety profiles regarding major postoperative complications, particularly severe hemorrhages requiring immediate surgical reintervention. Ultimately, the choice between these instruments depends on institutional availability and individual surgeon preference. Crucially, from a lymphatic standpoint, there is no definitive evidence suggesting that the type of energy device influences the baseline incidence of cervical chylous fistula; intraoperative safety and thoracic duct preservation remain fundamentally dependent on the surgeon’s meticulous anatomical awareness and tactile precision during level IV dissection, rather than the specific technology employed.

It is critical to note that while the pathophysiological mechanisms of thoracic duct injury remain identical across all surgical modalities, the recommendations and algorithms synthesized in this review apply primarily to conventional open lateral neck dissection. In minimally invasive, endoscopic, or robotic-assisted neck surgeries, the direct tactile feedback is replaced by magnified visual control. While robotic platforms offer superior visualization of level IV crossroads, the narrow surgical corridors can restrict multi-angle clip placement. Therefore, regardless of whether an open or a minimally invasive sequence is performed, strict adherence to visual anatomical preservation of the main duct remains the key preventative factor.

## 6. Clinical Appearance of Chylous Fistula

Unrecognized and unrepaired intraoperatively, cervical chylous fistula rapidly produces clinical signs and complications. The first and most common sign is copious fluid leakage from the drainage tubes. Serous-sanguinous fluid extravasated from the tubes in the thyroid lodge is common after thyroidectomy for 1–2 days. The milky-white appearance should attract attention. The volume of extravasated fluid may be another alarm sign [4]. Resumption of food, usually on the first day after surgery, leads to the absorption of nutrients, including lipids, from the intestine, and implicitly to an increase in lymphatic flow through the thoracic duct. A possible thoracic duct fistula will have a high flow and a milky, creamy appearance. In the case of a right lymphatic duct lesion, the appearance is clear (does not contain lipids), but the volume of fluid discharged into the drainage tube is excessive. This is why placing two drainage tubes (right and left) is better than using a single tube for both sides.

### 6.1. Diagnostic Evaluation and Identification of Fistula

Differentiating a true chylous fistula from ordinary postoperative lymphatic drainage or a seroma is critical for early therapeutic planning. Clinically, a macroscopic milky-white appearance is strongly suggestive, but can be masked during fasting or prior to the reintroduction of oral lipids. Thus, objective biochemical verification is mandatory. A fluid triglyceride concentration exceeding 100–110 mg/dL (or a level significantly higher than concurrent serum triglycerides) strongly supports a chyle leak. Conversely, ordinary lymphatic drainage features low triglyceride concentrations and remains clear regardless of diet. The absolute gold standard for diagnosis is lipoprotein electrophoresis demonstrating the presence of chylomicrons, which are completely absent in standard peripheral lymph. Furthermore, a dietary challenge (oral fat loading) triggers a dramatic surge in flow rate and turbidity exclusively in chylous fistulas. To facilitate rapid differential diagnosis, a comprehensive comparative matrix of postoperative fluid collections in the neck is detailed in Table 2.

Very seldom, when a lymphatic fistula is associated with a pleural lesion, chylothorax may occur [1]. Pleural effusion can be the consequence of surgery (damage to the pleural dome) or the pressure exerted by the leaked chyle in the chest.

The severity of the fistula is assessed according to its output: a low-output fistula is below 500 mL/day, a high-output fistula is 500 mL–1000 mL/day, and a massive fistula is over 1000 mL/day [3,49]. In a low-flow fistula there is a risk of metabolic imbalances (hypoproteinemia, hyponatremia, hypochloremia), but these are not severe. High-flow fistulas can quickly lead to dehydration, hypoproteinemia and lymphocytopenia. A massive fistula has a risk of major complications: dehydration, hypoproteinemia, lymphocytopenia, chylothorax, and death.

**Table 2:** Differential diagnosis matrix for postoperative cervical fluid collections.

Diagnostic Criterion	True Chylous Fistula	Ordinary Lymphatic Leak/Seroma	Abscess/Hematoma (Differential)
Macroscopic Appearance	Milky-white, creamy, or opalescent	Clear, straw-colored, or serosanguinous	Purulent (turbid yellow/green) or frankly bloody
Triglyceride Levels [3,50,51]	Elevated (>100–110 mg/dL) or >serum level	Low (<50 mg/dL); lower than serum levels	Low or variable; mirrors cellular debris values
Chylomicrons [3,51]	Present (gold standard via electrophoresis)	Absent or undetectable	Absent
Response to Oral Fat Test [3,49,51]	Massive increase in output and turbidity	No effect on volume or fluid clarity	No effect
Typical Daily Output	Highly variable; can easily exceed 500–1000 mL/d	Typically low (<100–150 mL/d) and steadily decreasing	Variable; self-limiting once evacuated

The flow rate not only indicates the severity of the lesion, but also the likelihood of spontaneous closure. Leović’s classification proposes other thresholds: below 300 mL/day for low-flow fistula, 300–600 mL/day for medium-flow fistula, and above 600 mL/day for high-flow fistula [51]. The threshold of 1000 mL/day is considered defining for the therapeutic strategy: below 1000 mL/day conservative treatment can be considered, but if the flow is above 1000 mL/day surgical treatment or interventional radiology is necessary [51].

When critically evaluating these two classification systems, Delaney’s threshold of 1000 mL/day demonstrates superior clinical and predictive value for modern surgical decision-making. While Leović’s lower threshold (600 mL/day) is sensitive for identifying patients at risk of gradual nutritional depletion, Delaney’s 1000 mL/day baseline serves as a reliable surrogate marker for major structural disruption of the main thoracic duct trunk. An unyielding output above this volume during the first 72 h carries an extremely low probability of spontaneous closure and carries an immediate threat of acute metabolic and electrolyte collapse. Consequently, operationalizing the 1000 mL/day rule as a hard ceiling—rather than waiting for a prolonged 14-day conservative trial—minimizes institutional stay and prevents severe patient degradation, justifying its role as the primary escalation trigger in our clinical algorithm.

### 6.2. Prognostic Impact and Clinical Course

The clinical prognosis and the socioeconomic burden of cervical chylous fistula are strictly correlated with the daily lymphatic output.

The low-output fistulas (<300–500 mL/day) typically present a benign course. While they do increase the hospitalization burden by requiring strict dietary monitoring, specialized enteral formulas, or prolonged drain maintenance (often extending hospital stay by 5 to 14 days), they rarely cause life-threatening systemic morbidity. Local wound morbidity is minimal if drainage is effective.

Ţhe high-output and massive fistulas (>500–1000 mL/day) carry a severe prognosis if not aggressively managed. The rapid, continuous loss of chyle induces acute hypovolemia and critical electrolyte depletion within 48–72 h. Over a longer timeframe, persistent lymphopenia severely impairs cellular immunity, predisposes the patient to secondary systemic infections, and delays wound healing. Local collection accumulation carries a high risk of skin flap necrosis and catastrophic erosion of adjacent major cervical vessels. Consequently, high-output fistulas significantly escalate morbidity, often requiring intensive care monitoring, parenteral nutrition support, or urgent surgical reintervention, substantially prolonging hospitalization and increasing overall healthcare utilization.

## 7. Treatment of Cervical Chylous Fistula

When critically evaluating the literature on cervical chylous fistula management, a significant methodological limitation becomes apparent: the absolute majority of published data relies on retrospective institutional case series, small observational cohorts, or isolated case reports. High-quality, prospective randomized controlled trials are exceptionally rare due to the low incidence of this complication. Consequently, current treatment hierarchies and consensus thresholds (such as the 1000 mL/day rule) are predominantly guided by expert opinion and retrospective data rather than high-level standardized evidence. Table 3 provides a comparative synthesis of these strategies, highlighting their clinical efficacy alongside the nature of the supporting evidence.

The treatment of cervical chylous fistulas is determined based on the daily volume of drainage and clinical progression.

For fistulas with low flow, conservative treatment can be attempted. This aims to decrease the fistula flow in order to close the defect in the lymphatic wall. For this, a diet is instituted from which long-chain fats are eliminated (animal fats, common vegetable oils, whole dairy products, butter, cheeses) because they are absorbed predominantly through the lymphatic system. Instead, medium-chain fats (coconut oil, palm oil) are administered because they do not form chylomicrons and are absorbed into the portal blood [3,51,52,53]. Another option is total parenteral nutrition [54,55], but this has several disadvantages: prolonged venous catheterization and catheter infection, atrophy of the intestinal mucosa, metabolic imbalances and high cost.

Octreotide decreases pancreatic and biliary secretion, both of which are involved in lipid digestion. This decreases the digestive absorption of lipids and the volume of chyle [50]. Another proposed mechanism is the alteration of gastrointestinal blood flow [56]. There is currently no international consensus or standardized guideline regarding the optimal dosing regimen for octreotide in cervical chyle leaks. This wide variation in reported dosing (100 to 1500 μg/day) stems from the empirical extrapolation of protocols long established in abdominal surgery for pancreatic and biliary fistulas. In clinical practice, the specific dosage for each patient is dynamically tailored based on two primary factors: the baseline output volume of the fistula and the immediate clinical response within the first 24–48 h. Typically, low-output leaks are managed with lower subcutaneous doses (e.g., 100 μg three times daily), whereas borderline or high-output cases often mandate high-dose continuous intravenous infusions (up to 500 μg three times daily or continuous pump delivery), with the dose titrated upward if the drainage volume fails to show a downward trend [51,57,58,59].

Applying compression dressings and maintaining drainage complement conservative treatment. Alternatively, negative pressure wound therapy can be used, with recommendations ranging from low-pressure configurations (−75 to −125 mmHg) to high-pressure settings (up to −600 mmHg) [60,61,62]. Recent clinical evidence strongly advocates for the utilization of low-pressure systems (continuous or intermittent settings around −125 mmHg) in the cervical region, while high-pressure systems might theoretically accelerate tissue granulation, they carry a catastrophic risk of major hemorrhage via mechanical erosion of adjacent major neck vessels, as emphasized in our summary matrix [51].

Injection of sclerosing agents into the drainage tube is another option for low-flow fistulas. Tetracycline, doxycycline, povidone-iodine (Betadine), or OK-432 (Picibanil) induces a fibrous reaction that can obstruct the fistula. Before the procedure, an ultrasound or X-ray is taken to check the position of the end of the drainage tube; then the substance is instilled into the tube and the tube is clamped. The procedure is often associated with pain, and the diffuse inflammation produced can have consequences for the vagus nerve or the phrenic nerve [63,64,65,66]. However, the method demonstrated a clinical success rate of up to 91% for low-flow fistulas in a retrospective cohort focused on post-dissection lymphoceles, primarily representing complete clinical resolution without surgical revision [64].

The effectiveness of conservative treatment is assessed by observing the volume and appearance of the drainage fluid, determining the triglycerides in the drainage fluid, and evaluating the clinical and nutritional stability of the patient. The most important indicator of a favorable evolution is the progressive decrease in the volume of fluid eliminated through the drain tube. A positive response is often observed within the first 24–48 h. Treatment is considered effective if it leads to complete cessation of the leakage within 5 to 14 days, thus avoiding surgical intervention.

With the decrease in volume, the appearance of the drainage also changes. If initially the fluid is milky and opalescent, as the lipid concentration decreases, it becomes serous and transparent. Repeated determination of the triglyceride concentration shows, in the case of effective treatment, their gradual decrease, reaching below 110 mg/dL. The absence of chylomicrons in the biochemical analysis of the fluid confirms the success of the therapy. Maintenance or normalization of protein (albumin) and electrolyte levels in the blood indicates that the losses through the fistula are no longer clinically significant. After the disappearance of the drainage, the normal diet including long-chain lipids is reintroduced. If the drainage does not reappear, the fistula is considered closed. Persistence of drainage without a tendency to decrease for more than 3 days or persistence of a fistula beyond 10–14 days suggests failure of conservative treatment and grants reconsideration of the treatment options [3,49,50,51,52,53].

If drainage exceeds 1000 mL/day or if conservative treatment does fails after 7–14 days, surgical or interventional treatment is required.

To identify the duct and fistula during surgery, hyperlipidic nutrition administered the day before surgery or intraoperative lymphangiography can be used. The administration of foods rich in long-chain fatty acids increases the volume of lymph and makes the leakage more obvious due to the milky appearance of the chyle rich in such fatty acids. After opening the wound, washing and drying the surgical field, the patient is tilted in the anti-Trendelenburg position and the Valsalva maneuver is performed by closing the Adjustable Pressure Limiting (APL) valve of the anesthesia machine, performing continuous positive pressure ventilation and maintaining a constant pressure of about 40 cm of water for 15–20 s. Identification of the fistula by indocyanine green lymphography is the second option. Usually the duct is visualized in the jugulo-subclavian venous angle, but anatomical variations can cause it to be discovered in other positions. After identifying the fistula, ligation is performed with non-absorbable suture. Given the fragility of the duct, the ligation must also include adjacent tissues to prevent tearing of the duct. However, this increases the risk of damage to adjacent anatomical structures (phrenic nerve, recurrent nerve, vagus nerve, subclavian artery); these structures must be carefully protected.

For additional sealing of the thoracic duct ligation, tissue adhesives, cyanoacrylate (N-2-butyl cyanoacrylate), collagen sponges, microfibrillar collagen, or collagen sponges with fibrin and thrombin can be used [49,67]. Local muscle flaps using the omohyoid muscle or a pedicled flap from the inferior clavicular portion of the sternocleidomastoid muscle fixed to the carotid fascia and prevertebral fascia create a tight barrier and compress the thoracic duct between the flap and the prevertebral muscles [68,69,70,71]. Combining the application of sealants with the fixation of a muscle flap is also an option [6], after which a Valsalva maneuver is performed to verify the effectiveness of the seal. The suction drain placed at the end of the surgery is essential for monitoring the volume and appearance of the evacuated chyle and for evaluating the effectiveness of the operation.

Thoracoscopic thoracic duct ligation or percutaneous duct embolization are the next treatment options if the cervical approach fails. Thoracoscopic ligation requires selective intubation of the left bronchus and collapse of the right lung. Trocars are inserted in the 7th or 8th intercostal spaces in the posterior axillary line (video camera), and 5th or 6th and 8th or 9th intercostal spaces in the midaxillary line or anterior axillary line (working instruments). The thoracic duct is identified in the posterior mediastinum, between the azygos vein and the aorta, and is sealed with 1–2 clips. The possibility of the existence of two ducts or other anatomical variants is also considered [72,73].

Thoracic duct embolization begins with intranodal or pedal lymphangiography that ensures the identification of the cisterna chyli. The cisterna chyli is approached by percutaneous puncture, then the catheter is inserted along a guidewire. Obstruction of the duct is done with a mixture of micro-coils (small metal coils), cyanoacrylate and lipidiodol. The method has a technical success rate of 70–90% regarding successful cisterna chyli cannulation and occlusion, translating to a clinical success rate of approximately 75–85% in resolving high-output cervical leaks without subsequent open surgery [74,75,76,77]. The metal coils (micro-coils) and the duct valves limit the obstruction to a limited duct segment. Dilation of the small lymphovenous anastomoses at the upper chest level ensures lymphatic drainage after a while. However, in the case of multiple and small-caliber ducts, cannulation and embolization are not possible [74,75,76,77,78,79].

The effectiveness of the treatment is assessed by monitoring the drainage and by the lipid loading test (“the fat challenge”). A decrease in the drainage volume to 30–50 mL/day and its clear aspect suggest an effective sealing of the duct/fistula. After 24–48 h of minimal drainage, long-chain lipids (cream, oil) are administered orally. If the drainage remains reduced and clear and does not change to voluminous and milky, the fistula is considered closed. Determination of the concentration of triglycerides in the drainage fluid or electrophoresis in search of chylomicrons can also be used [3,49,51,52].

After the drainage is removed, several simple measures are taken to ensure permanent healing: gradual transition (over about 3 weeks) to a common diet, avoidance of activities that involve physical exertion and increase intrathoracic pressure, adoption of a Fowler position during periods of rest and sleep, restriction of smoking (if applicable) and treatment of cough [3,49,50,51,52].

Treatment options, with their indications, advantages and disadvantages, are presented in Table 3.

**Table 3:** Comparative synthesis of therapeutic options in cervical chylous fistula.

Therapeutic Option	Clinical Indication	Reported Success Rate	Strength of Evidence/Study Types	Main Advantages	Limitations/Risks
Dietary Modification (MCT/Low-fat)	First-line for low-output fistulas (<300–500 mL/day)	~50–70% clinical success (as monotherapy) [3,51,58]	Moderate (Retrospective cohorts, institutional guidelines)	Non-invasive, easy to implement	Patient non-compliance, high failure rate in high-output leaks
Octreotide Therapy	Adjuvant to dietary changes in low/medium-output fistulas	>80% clinical success (when combined with diet) [57,58,59,80]	Moderate to High (Prospective studies, systematic reviews)	Rapid flow reduction (24–48 h), shortens hospital stay	Side effects (nausea, cramps), lack of standardized dosage
Negative Pressure Wound Therapy	Refractory low-output or selected medium-output fistulas	Highly variable ~70–90% clinical success in small series [60,61,62]	Low (Case reports, small case series)	Accelerates tissue granulation and skin flap adherence	Risk of major hemorrhage via erosion of major neck vessels
Sclerotherapy	Persistent low-output fistulas failing initial diet/octreotide	Up to 91% clinical success [64]	Low to Moderate (Retrospective series)	Minimally invasive, high success in localized lymphoceles	Local pain, severe diffuse inflammation, risk of phrenic/vagus nerve injury
Surgical Reintervention (Ligation/Flaps)	High-output (>1000 mL/day) or failed conservative treatment	High (definitive closure if duct identified) [68,69,70,71]	Moderate (Retrospective surgical cohorts)	Definitive mechanical closure of the leak	Difficult dissection due to local inflammation, risk of nerve injury
Thoracic Duct Embolization (TDE)	Failed cervical surgery or primary choice for high-output leaks	70–90% technical success, 75–85% clinical success [74,75,76,77]	Low to Moderate (Retrospective interventional series)	Avoids re-entering a hostile, inflamed neck surgical field	Requires advanced technical expertise and specialized equipment

## 8. Proposed Evidence-Informed Framework for Clinical Escalation

**Figure 6:**
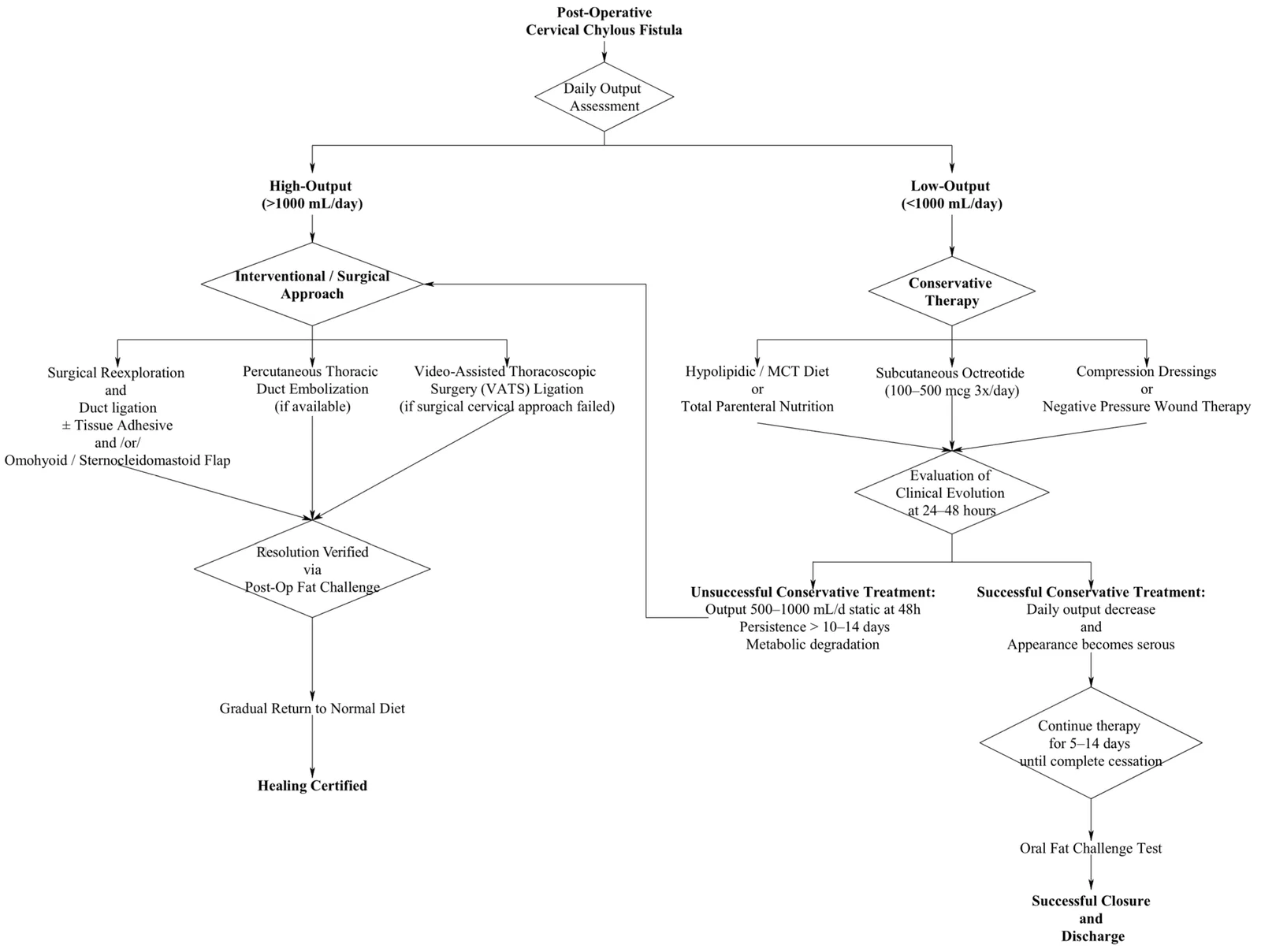
Proposed evidence-informed framework for clinical escalation. Note: All output measurements and threshold criteria are based on cumulative 24-h drain volumes, representing the sum of both left and right cervical drains when bilateral drainage is utilized.

To provide practical clinical guidance, the management of post-thyroidectomy chylous fistula is proposed here as a structured, evidence-informed framework based on daily output and clinical evolution (Figure 6):

1. Expected timeframe for response: a positive response to conservative management (hypolipidemic/MCT diet and octreotide) should ideally manifest within the first 24 to 48 h, characterized by a progressive, steady decline in drainage volume and a transition from milky to serous fluid.
2. Criteria for treatment failure: conservative management is considered to have failed if:
  - The daily drainage remains sustained above 1000 mL/day for more than 3 days despite optimal fasting/dietary and medical therapy.
  - The drainage does not show a downward trend or fail to cease completely within 10 to 14 days [3,49,50,51,52,53].
  - The patient develops rapid, severe nutritional degradation (refractory hypoproteinemia) or metabolic/electrolyte imbalances that cannot be compensated.

All daily output thresholds specified in this framework refer strictly to the cumulative 24-h drainage volume, representing the sum of both left and right cervical drains when bilateral drainage is utilized.

Upon defining treatment failure, immediate escalation is mandatory. For border-line or high-output leaks (500–1000 mL/day) with metabolic decline, surgical re-ligation (with muscle flaps/sealants) is the primary choice, while percutaneous embolization or thoracoscopic ligation serve as a definitive option for surgical failures or hostile neck environments.

## 9. Discussion

Aspects of cervical lymphadenectomy relevant to the risk of chylous fistula include protecting the thoracic duct and right lymphatic duct during dissection of the lower neck nodes. The right lymphatic duct can be a source of persistent lymphadenopathy, but this is an extremely rare complication [81]. More frequently (even though it remains a rare complication) persistent chylous fistula following lymphadenectomy for thyroid carcinoma is at the level of the thoracic duct.

The risk arises during dissection of level IV (inferior jugular nodes), where the thoracic duct (on the left side) and the right lymphatic duct enter the jugulosubclavian venous confluence. A key element in preventing complications, including thoracic duct lymphatic fistula, is to take only necessary risks and avoid unnecessary risks and procedures of uncertain therapeutic benefit. The best way to avoid injury to the thoracic duct is to avoid dissection at the base of the neck on the left side unless absolutely necessary [10].

Lateral compartment lymphadenectomy is performed in all cases when there is nodal metastasis in this space. If there is no evidence of metastasis in the lateral compartment, the indication should take into consideration the type of tumor, the size and location of the tumor, the suspicion of multicentricity, the number of metastatic nodes in the central compartment, and the patient’s age, as well as subtle ultrasound signs of central node involvement [23,24,25,26,27,28,29]. Chylous fistula, however rare a complication, occurs predominantly in the case of lateral compartment lymphadenectomy, more precisely during the removal of the inferior jugular nodes (group IV). Nonetheless, safe clearance of the central compartment (level VI) can occasionally trigger lymphatic leaks or, in exceptional cases of anomalous or ectopic thoracic duct micro-branches, high-output chylous fistulas [1,5]. On the other hand, reintervention has a higher complication rate [82].

Knowledge of the cervical trajectory and anatomical variants of the duct is essential for the prevention of fistula. The opening of the duct into the venous system is variable because it can open into the internal jugular vein, the subclavian vein, the angle between these, or into other veins. Also, the termination of the canal can have two, three, or four branches and can receive up to three tributaries [34,35,36,37,38]. The arch that the duct describes above the clavicle is usually 3–4 cm high, but it can be higher [44]. All of these variations, along with the thin and fragile walls of the duct, are risk factors for damage during surgery.

When performing group IV lymphadenectomy, the surgeon must be aware of the variable anatomy of the thoracic duct. It is preferable to identify the duct and possibly mark its position with a loop placed nearby. The Valsalva maneuver can be used for identification, but the best method is indocyanine green lymphography [45,46]. If thoracic duct ligation is necessary (to include the tissue at the terminal portion of the internal jugular vein in the dissection), additional tissue should be included in the ligation to avoid sectioning the thin wall of the thoracic duct with the ligature [10]. Dissection in the opposite direction, along the lymphatics discovered in the adipose tissue from the jugulo-subclavian venous angle, may be a variant of discovering and protecting the thoracic duct and the right lymphatic duct [47].

Checking the integrity of the thoracic duct before completing the operation is mandatory. Sometimes the surgeon has the sensation that the gloves have become sticky [83]. After placing the patient in the anti-Trendelenburg position and drying the surgical field, the Valsalva maneuver is performed. The leakage of a clear, yellowish fluid indicates the lesion and with attention the source of the lymphorrhage can also be identified. If indocyanine green lymphography is performed, a final examination in infrared light is useful.

In the case of a ruptured duct, the ligation is performed including additional tissue in the ligature. If the damaged duct cannot be identified, a ligature is performed including a consistent portion of tissue [10]. However, the method has the risk of damaging neighboring structures (phrenic nerve, recurrent nerve, stellate ganglion) and therefore must be performed carefully. Then the Valsalva maneuver is repeated and the surgical field is carefully examined.

Although the right lymphatic duct is rarely a problem, the same principles should be applied in the case of right chylous drainage [10].

Loss of chyle can lead over time to hypoproteinemia, hyponatremia, and dehydration.

The loss of lymphocytes, especially T lymphocytes, can be followed by lymphopenia and immunodepression [83].

The existence of several fistula classification systems based on flow rate shows the difficulty of therapeutic decisions [1,3,49,50,51]. The higher the flow, the lower the probability of its closure spontaneously or under conservative treatment.

The objective of conservative treatment of chylous fistula is to decrease the production of chyle to create conditions for healing of the fistulous opening. The central pillar of this objective is the drastic reduction in the oral intake of long-chain lipids. These lipids form chylomicrons and are absorbed into the lymph. Chylous fistula leads to massive loss of proteins, calories and fat-soluble vitamins (A, D, E, K), with the risk of malnutrition and severe hydroelectrolytic imbalances. To prevent such complications, the diet must be hypolipidemic and hyperproteinemic. Medium-chain lipids can be administered, since they do not form chylomicrons and do not increase the volume of lymph absorbed from the intestine. Oral nutrition can be supplemented with parenteral nutrition. However, total parenteral nutrition is often insufficient to prevent malnutrition, has side effects and is expensive [3,51,52,53,54,55].

The diet is extremely restrictive, under 20–25 g of fat per day. Available guidelines can be used, for example Nova Scotia Health or Alberta Health Services [83,84,85]. Standardized preparations are available either as complete enteral formulas with low long-chain triglycerides and high short-chain triglycerides (Monogen, Nutrison MCT, Peptamen), or as modular supplements containing short-chain fatty acid oils (Liquigen) [86,87].

The mechanism of action of octreotide is indirect by decreasing blood flow to the abdominal viscera, by inhibiting digestive secretions involved in the digestion and absorption of lipids and by direct action on vascular receptors for somatostatin. But even acting indirectly, octreotide is the most important adjuvant to dietary treatment, the clinical success rate of complete fistula closure being over 80% when combined with structural low-fat diets in head and neck cancer populations [57,58,59,80]. The combination of severe lipid-restricted nutrition and octreotide leads to a reduction in fistula flow within 24–48 h and closure of low-flow fistulas within 5–7 days [59]. There is no consensus on the recommended dose.

Negative pressure therapy and sclerosing therapy complement diet and octreotide treatment. The combination of the aforementioned methods ensures a success rate of over 90% in the case of low-flow fistulas [64].

When utilizing negative pressure wound therapy in a hostile, inflamed neck environment, prioritizing low-pressure continuous suction (around −125 mmHg) is critical to protect the carotid wall from dynamic shear stress and prevent arterial wall necrosis [51].

Careful monitoring of the patient should identify in a timely manner cases in which conservative treatment is ineffective. Prolonging conservative treatment does not bring any benefit to the patient if a rapid decrease in fistula flow is not observed. Instead, it exposes the patient to the occurrence of complications. Changing the therapeutic strategy should be considered at any time during treatment. A 24–48 h re-evaluation window and the 10–14-day escalation ceiling represent a pragmatic, evidence-informed proposal aimed at balancing two critical surgical risks: avoiding an unnecessary, technically challenging reoperation in a highly inflamed surgical bed versus preventing refractory metabolic and nutritional degradation. Prolonging conservative management in a patient with unyielding high-volume drainage significantly alters cellular immunity and tissue healing, forcing a subsequent surgical rescue under far worse biological conditions.

Clinical and laboratory criteria are simple (volume of drained fluid, aspect of the fluid, aspect of the wound, triglyceride level in the fluid, patient weight, protein and albumin levels, ionogram and lymphocytes), but they must be checked rigorously. Conservative treatment is effective if the volume of the drainage decreases significantly within 3 days and the fistula closes within 10–14 days. Failure to meet these criteria requires reconsideration of treatment options [3,49,50,51,52,53].

Reopening the wound and ligation of the duct are the most feasible options when conservative treatment fails or when the fistula has a high flow rate. However, identification of the duct can be difficult due to a combination of factors: inflammation, duct fragility (which is usually collapsed), highly variable anatomy, and transparency of the lymph after stopping lipid feeding. Administration of fats on the day before surgery or 5–6 h before (orally or via nasogastric tube) and an intraoperative Valsalva maneuver help to identify the fistula. Indocyanine green lymphangiography may be necessary.

Duct ligation must take into account the fragility of the duct and the presence of inflammation. The ligature thread can cut the fragile duct, and the operation is ineffective. As a result, the ligature must also capture adjacent tissues. In addition, tissue adhesives, collagen or local muscle flaps can be used for coverage. A Valsalva maneuver to certify the tightness of the ligature is indicated [49,67,68,69,70,71].

Thoracoscopic ligation or thoracic duct embolization are both indicated in cases where surgical cervical ligation fails and as an initial treatment method.

Thoracoscopic ligation has the advantage of intervening on a healthy duct segment, unaffected by inflammation in the neck, but requires a multidisciplinary team (thoracic surgeon) and a general condition of the patient that allows selective pulmonary intubation [72,73]. Thoracic duct embolization is effective, but requires advanced technology and specialized personnel, which are not always available.

Regardless of the treatment method used, the test of effectiveness is given by resuming the usual diet, containing long-chain lipids.

Ultimately, while cervical chylous fistula represents a highly specific and rare subset of lymphatic morbidity, its preventive and escalation strategies should be integrated into the broader global framework of classic complications in thyroid surgery and their comprehensive contemporary management [48].

### Current Knowledge Gaps and Future Research Directions

Despite the clinical importance of cervical chylous fistula, this comprehensive synthesis highlights a critical framework gap in the current literature: the complete absence of multi-center, prospective randomized controlled trials. Current clinical algorithms, including the widely utilized 1000 mL/day escalation threshold, are fundamentally anchored in expert opinions and retrospective institutional cohort data. Future clinical research should focus on three main gaps: first, establishing standardized, multi-center prospective databases to validate volume-based escalation algorithms; second, conducting comparative-effectiveness trials to define the optimal dosing and timing criteria for somatostatin analogues (octreotide); and third, performing objective cost–benefit analyses evaluating early percutaneous embolization versus traditional surgical re-ligation in borderline high-output leaks. Addressing these gaps is paramount to transitioning chylous fistula management from empirical consensus to high-level, evidence-based guidelines.

## 10. Conclusions

Cervical chylous fistula occurs following dissection of the lateral compartment, specifically the supraclavicular nodes (group IV). The indication for the type of lymphadenectomy must take into account the type of thyroid carcinoma, the imaging evidence of lymph node dissemination, and also the risk of complications. Given the severity of the consequences of the fistula, it must be recognized and treated promptly. The main therapeutic decision is the choice between conservative and interventional treatment (surgical or radiological). This decision is based on the volume of fistula flow; the chances of success of conservative treatment are lower in cases of higher fistula flow. Although individual clinical contexts vary, conservative management is highly effective for low-output fistulas (<500 mL/day), whereas borderline outputs (500–1000 mL/day) require a strict time-bound trial of 24–48 h with a low threshold for escalation. An output exceeding 1000 mL/day, or a lack of response to conservative therapy, constitutes a definitive indication for interventional escalation, including surgical re-ligation, thoracoscopy, or percutaneous embolization, as detailed in our clinical algorithm. Careful monitoring is necessary for timely identification of cases in which conservative treatment is ineffective and switching to more aggressive treatment methods is necessary.

## Figures and Tables

**Table 1:** Types of lymphadenectomy.

Neck Level/Compartment	Anatomical Boundaries (Superior/Inferior/Lateral)	Surgical Dissection Type in Thyroid Cancer	Relevance to Chylous Fistula Risk
Level VI (Central)	Hyoid bone/Sternal manubrium/Carotid arteries	Central Neck Dissection (CND): Bilateral or unilateral removal of pretracheal, paratracheal, and prelaryngeal nodes.	Low risk; potential injury to anomalous micro-branches.
Levels II, III, IV (Lateral)	Skull base/Clavicle/Posterior border of Sternocleidomastoid muscle	Selective Neck Dissection (SND Levels II–IV): Standard therapeutic clearance for lateral metastases (\(N1b\)).	Critical Risk Zone: Level IVB (inferior jugular) matches the anatomical confluence of the thoracic duct.
Level V (Posterior Triangle)	Convergence of SCM and Trapezius/Clavicle/Anterior border of Trapezius	Extended Lateral Dissection: Dissection of VA (cranial) and VB (caudal) divided by the omohyoid muscle.	Moderate risk at Level VB near the transverse cervical lymphatic branches.

## Data Availability

No new data were created or analyzed in this study. Data sharing is not applicable to this article.
